# Ambulatory pulse oximetry monitoring in Japanese COPD outpatients not receiving oxygen therapy

**DOI:** 10.1186/2049-6958-9-24

**Published:** 2014-04-17

**Authors:** Seigo Minami, Suguru Yamamoto, Yoshitaka Ogata, Takeshi Nakatani, Yoshiko Takeuchi, Masanari Hamaguchi, Taro Koba, Kiyoshi Komuta

**Affiliations:** 1 Department of Respiratory Medicine, Osaka Police Hospital, 10-31 Kitayama-cho, Tennoji-ku, Osaka 543-0035, Japan; 2 Department of Internal Medicine, National Hospital Organization Kinki-Chuo Chest Medical Center, 1180 Nagasone-cho, Kita-ku, Sakai, Osaka 591-8555, Japan; 3 Department of Respiratory Medicine, Allergy and Rheumatic Diseases, Osaka University Graduate School of Medicine, 2-2 Yamada-oka, Suita, Osaka 565-0871, Japan; 4 Osaka Prefectural Medical Center for Respiratory and Allergic Diseases, 3-7-1 Habikino, Osaka 583-8588, Japan

**Keywords:** Activities of daily living, Chronic obstructive pulmonary disease (COPD), Desaturator, Exacerbation, Outpatients, Pulse oximetry

## Abstract

**Background:**

It remains unknown whether desaturation profiles during daily living are associated with prognosis in patients with chronic obstructive pulmonary disease (COPD). Point measurements of resting oxygen saturation by pulse oximetry (SpO_2_) and partial pressure of arterial oxygen (PaO_2_) are not sufficient for assessment of desaturation during activities of daily living. A small number of studies continuously monitored oxygen saturation throughout the day during activities of daily living in stable COPD patients. This study aims to analyse the frequency of desaturation in COPD outpatients, and investigate whether the desaturation profile predicts the risk of exacerbation.

**Methods:**

We studied stable COPD outpatients not receiving supplemental oxygen therapy. Baseline assessments included clinical assessment, respiratory function testing, arterial blood gas analysis, body mass index, and the COPD Assessment Test (CAT). Patients underwent 24-hour ambulatory monitoring of SpO_2_ during activities of daily living. Exacerbations of COPD and death from any cause were recorded.

**Results:**

Fifty-one patients were enrolled in the study, including 12 current smokers who were excluded from the analyses in case high serum carboxyhaemoglobin concentrations resulted in inaccurately high SpO_2_ readings. The mean percent predicted forced expiratory volume in one second (%FEV_1_) was 50.9%. The mean proportion of SpO_2_ values below 90% was 3.0% during the day and 7.4% during the night. There were no daytime desaturators, defined as ≥ 30% of daytime SpO_2_ values below 90%. Twenty-one exacerbations occurred in 13 patients during the mean follow-up period of 26.4 months. Univariate and multivariate Cox proportional hazards analyses did not detect any significant factors associated with exacerbation.

**Conclusions:**

Our 24-hour ambulatory oximetry monitoring provided precise data regarding the desaturation profiles of COPD outpatients. Both daytime and nighttime desaturations were infrequent. The proportion of ambulatory SpO_2_ values below 90% was not a significant predictor of exacerbation.

## Background

Chronic obstructive pulmonary disease (COPD) is a leading cause of morbidity and disability worldwide, and is predicted to become the third highest cause of death by 2020 [1]. Desaturation profiles during a 6-minute walk test (6MWT) may predict prognosis primarily in patients with severe COPD with a percent predicted forced expiratory volume in one second (FEV_1_) of < 50% [2]. Time to desaturation during a 6MWT also predicts desaturation time in 24-hour ambulatory oximetry monitoring primarily in moderately hypoxaemic COPD patients with a resting partial pressure of arterial oxygen (PaO_2_) between 60 and 70 mmHg [3]. However, it remains unknown whether desaturation profiles measured by ambulatory oximetry can predict prognosis in COPD patients. Transient desaturations have been observed in patients with moderate to severe chronic pulmonary disorders, even without significant resting hypoxemia [4]. Point measurements of resting oxygen saturation by pulse oximetry (SpO_2_) and PaO_2_, the conventional parameters used to determine requirements of long-term oxygen therapy, are not sufficient for assessment of desaturation during activities of daily living [5]. Field walking tests such as the 6MWT, which is the standard test used for assessment of functional exercise tolerance, do not always provide a good reflection of variations in oxygen saturation, because most activities of daily living are performed at submaximal levels of effort. 6MWT results did not predict the degree of desaturation during defecation in patients with chronic respiratory failure [6]. Conventional assessment methods are therefore not satisfactory for obtaining a comprehensive understanding of oxygen saturation throughout the day.

Pulse oximetry is a rapid, non-invasive method of monitoring the oxygen saturation of haemoglobin. Recent technological advances have enabled development of low-cost, small, user-friendly, portable pulse oximeters that can record data for over 24 hours. Many studies have evaluated the usefulness of pulse oximetry in COPD patients, but most studies only monitored patients continuously during the night or for a short period during the day. Although several studies monitored patients continuously during the day, most of these included patients with severe COPD on long-term oxygen therapy, or exclusively analysed patients on long-term oxygen therapy.

This study aimed to evaluate the desaturation profiles of Japanese COPD outpatients not receiving supplemental oxygen therapy during activities of daily living at home using continuous 24-hour pulse oximetry monitoring, and analyse the frequency of desaturation and relationships between the desaturation profile and the clinical characteristics and risk of exacerbation. The ability of the desaturation profile to predict exacerbation of COPD was investigated.

## Methods

### Patient selection

Patients were recruited from the outpatient clinic in the Department of Respiratory Medicine at Osaka Police Hospital. The inclusion criteria were: (1) clinically stable COPD according to the Global Initiative on Obstructive Lung Disease guidelines, with a FEV_1_ to forced vital capacity ratio of < 70% and minimal or no reversibility to β_2_-agonists (<200 mL and/or < 15%); (2) able to walk independently or with a cane; and (3) sufficient cognitive function to follow the instructions for using the pulse oximeter. Patients were excluded if they were currently hospitalised, if they had experienced an exacerbation requiring hospitalisation or administration of antibiotics or steroids during the preceding 3 months, if they had other significant respiratory diseases or medical problems that precluded ambulatory activities at home, and if they were receiving supplemental oxygen therapy.

### Experimental design

Within 1 month of ambulatory oximetry monitoring, all patients underwent spirometry, arterial blood gas analysis, assessment of the modified Medical Research Council (mMRC) dyspnoea scale score, and the Japanese version of the COPD assessment test (CAT) (GlaxoSmithKline; http://www.catestonline.org/) [7,8]. Spirometry was performed using an autospirometer (Autospirometer System 7; Minato Medical Science, Osaka, Japan) according to the American Thoracic Society guidelines. Samples for arterial blood gas analysis were obtained from the radial artery with the patient resting in the sitting position and breathing room air, and PaO_2_ and partial pressure of arterial carbon dioxide (PaCO_2_) were immediately measured using a blood gas analyser (RAPIDPoint 405 Arterial Blood Gas Analyzer; Siemens Healthcare Diagnostics Inc., Tarrytown, NY, USA).

A portable pulse oximeter (PULSOX-Me300; Konica-Minolta, Tokyo, Japan) was used to obtain continuous oximetry data over a 24-hour period. SpO_2_ and the pulse-rate waveform were recorded every second. A nurse individually instructed each patient on the proper use of the portable oximeter before the start of monitoring. The finger probe of the portable oximeter was positioned on the patient’s non-dominant hand to minimise interference during normal daily activities. The portable oximeter was attached to the patient’s wrist during the monitoring period. Periods during which the heart rate fell abruptly by ≥ 25 beats/minute were excluded from the analyses. All data were downloaded to a computer for analysis (DS-Me version 2.10). A calibration check according to the manufacturer’s instructions was performed before and after use. Patients were instructed to keep an activity log to record the times of major daily activities (sleeping, eating, walking outdoors, bathing, and other physical activities). Based on the patient’s activity log, periods of sleep were defined as night, and all other periods were defined as day. Patients were encouraged to perform all normal daily ambulatory activities, and to maintain their baseline levels of activity. The daytime and nighttime mean SpO_2_ value, proportion of SpO_2_ values below 90%, and heart rate were analysed. The 3% oxygen desaturation index (3%ODI) during the night was measured to detect patients with possible obstructive sleep apnoea (OSA) [9].

Patients were followed up for as long as possible after their ambulatory oximetry monitoring, and exacerbations and survival were analysed. Exacerbations were recorded from the day of oximetry monitoring. Follow-up ended on 30 November 2013. Exacerbation was defined as an increase in respiratory symptoms lasting at least 3 days and requiring treatment with antibiotics or systemic steroids.

The primary outcome was the proportion of desaturators, defined by Levi-Valensi *et al.* as patients with ≥ 30% of SpO_2_ values below 90% [10]. This definition was validated by Casanova *et al*. [11] and has also been used in other recent studies of 24-hour or daytime oximetry monitoring [3,5,12]. The secondary outcomes were the mean SpO_2_, heart rate and occurrence of exacerbation. Based on the results of previous studies [3,11], the proportion of desaturators was estimated to be approximately 24% in COPD patients with a resting PaO_2_ of 60–70 mmHg. The proportion of desaturators in this study was estimated to be 8%, because we expected the study to include a higher proportion of patients with mild COPD than previous studies. For a one-sided alpha of 5% and a power of 80%, we estimated that 47 evaluable subjects would be needed. Given the possibility of incomplete data in some patients, we considered that 50 subjects would be needed for this study. The study was approved by the Osaka Police Hospital ethics committee. All patients provided written informed consent for inclusion in the study.

### Data analysis

The values for normally distributed continuous variables, discrete variables, and categorical variables are expressed as the mean ± standard deviation (SD), median (range), and frequency, respectively. Comparisons between groups were performed using the unpaired *t*-test for normally distributed continuous variables. Simple and multiple regression analyses were performed to evaluate relationships between variables and the proportion of daytime SpO_2_ values below 90%. The following factors were included in the simple regression analyses: percent predicted FEV_1_ (%FEV_1_), mMRC dyspnoea grade, CAT score, resting PaO_2,_ resting PaCO_2_, body mass index (BMI), and age. All variables with a *p* value of < 0.2 on univariate analysis were included in the multiple regression analysis. The multiple regression analysis results are expressed as the unstandardized regression coefficient (β) with the corresponding 95% confidence interval, and the coefficient of determination (R^2^). Univariate and multivariate Cox proportional hazards models were used to evaluate relationships between the variables and exacerbation. The following factors were included in the multivariate analysis: age-adjusted Charlson Comorbidity Index [13], BMI, %FEV_1_, and proportion of SpO_2_ values below 90% during the day and night. The Cox proportional hazards analysis results are expressed as risk ratio (RR) with the 95% confidence interval. A two-tailed *p* value of < 0.05 was considered statistically significant. Statistical analyses were performed using StatMate statistical software (StatMate version IV; ATMS Co., Ltd., Tokyo, Japan). The sample calculation was performed using EZR (Saitama Medical Centre, Jichi Medical University, Saitama City, Japan), a graphical user interface for R (The R Foundation for Statistical Computing) [14]. EZR is a modified version of R commander designed to add statistical functions and frequently used in biostatistics.

## Results

A total of 51 Japanese patients were enrolled in the study from February 2011 to September 2011. Thirty-eight patients were ex-smokers, 12 were current smokers, and 1 was a non-smoker who had been chronically exposed to excessive environmental tobacco smoke. We excluded the 12 current smokers from our analyses, because smoking status was not considered at study entry and some current smokers were unexpectedly included. Current smoking may increase SpO_2_ values due to a high serum carboxyhaemoglobin (COHb) concentration [15-17]. The characteristics of the ex-/non-smokers are shown in Table 1.

**Table 1:** Baseline characteristics of ex-/non-smokers (n = 39)

Age (years)	Mean ± SD	71.6 ± 8.8
Sex	Male/female	36/3
Smoking pack/years	Mean ± SD	68.2 ± 33.4
BMI (kg/m^2^)	Mean ± SD	23.1 ± 3.8
Underweight (BMI < 18.5 kg/m^2^)	n (%)	4 (10.3)
Overweight (BMI 25–30 kg/m^2^)	n (%)	5 (12.8)
Obese (BMI ≥ 30 kg/m^2^)	n (%)	2 (5.1)
Unadjusted Charlson Comorbidity Index	Median (range)	1 (0–4)
Age-adjusted Charlson Comorbidity Index	Median (range)	5 (2–9)
Treatment		
Long-acting muscarinic antagonist	n (%)	26 (67%)
Long-acting β_2_ agonist	n (%)	27 (69%)
Inhaled corticosteroid	n (%)	27 (69%)
PaO_2_ (mmHg)	Mean ± SD	78.2 ± 8.9
PaCO_2_ (mmHg)	Mean ± SD	39.1 ± 4.9
GOLD stage	I/II/III/IV	2/18/13/6
FEV_1_ (L)	Mean ± SD	1.3 ± 0.6
FEV_1_ (%predicted)	Mean ± SD	50.9 ± 18.5
FVC (L)	Mean ± SD	2.3 ± 1.0
mMRC dyspnoea grade	Mean ± SD	2.2 ± 1.1
	Median (range)	2 (0–4)
CAT score	Mean ± SD	11.7 ± 7.0
	Median (range)	10 (0–29)

BMI, Body mass index; CAT, Chronic obstructive pulmonary disease assessment test; FEV_1_, Forced expiratory volume in one second; FVC, Forced vital capacity; GOLD, Global Initiative on Obstructive Lung Disease; mMRC, Modified Medical Research Council; SD, Standard deviation.

The daytime and nighttime oximetry monitoring results are shown in Table 2. There were no daytime desaturators and three nighttime desaturators. Although we did not confirm OSA by polysomnography, the results showed possible OSA (3%ODI of more than 15 desaturations/hour) in six patients [9]. Comparison of these 6 patients with the remaining 33 patients found no significant differences in the proportion of SpO_2_ values below 90% in the daytime (1.7 ± 2.1 vs 3.2 ± 6.0%, *p* = 0.28) or nighttime (15.4 ± 9.9 vs 5.9 ± 17.0%, *p* = 0.08) or in the mean daytime SpO_2_ value (94.6 ± 0.8 vs 94.7 ± 1.3%, *p* = 0.86), but did find a significant difference in the mean nighttime SpO_2_ value (92.3 ± 0.9 vs 93.9 ± 1.8%, *p* = 0.03). In the nine patients with a resting PaO_2_ of 60–70 mmHg (resting PaO_2_ 66.2 ± 2.8 mmHg), including no daytime and two nighttime desaturators, the proportion of SpO_2_ values below 90% was 8.4 ± 9.0% during the day and 20.2 ± 28.5% during the night, and the 3%ODI was more than 15 desaturations/hour in one ex-smoker (15.6 desaturations/hour).

**Table 2:** Pulse oximetry monitoring results in ex-/non-smokers (n = 39)

	**Daytime**	**Nighttime**
Monitoring duration (hours)		
Mean ± SD	14.5 ± 3.7	8.5 ± 3.0
Proportion of SpO_2_ values < 90% (%)		
Mean ± SD	3.0 ± 5.5	7.4 ± 16.1
Median (range)	1.1 (0.0–28.1)	1.3 (0.0–87.3)
Mean SpO_2_ (%)		
Mean ± SD	94.7 ± 1.2	93.7 ± 1.8
Median (range)	94.9 (90.7–97.4)	93.9 (88.8–96.8)
Heart rate (beats/min)		
Mean ± SD	76.9 ± 10.6	69.0 ± 9.0
Median (range)	75.0 (49.4–101.0)	68.2 (49.4–87.1)

SD, Standard deviation; SpO_2_, Oxygen saturation by pulse oximetry.

Simple regression analysis showed that higher %FEV_1_ (β = -0.12, *p* = 0.01), higher PaO_2_ (β = -0.30, *p* = 0.002) and lower mMRC dyspnoea grade (β = 2.38, *p* = 0.002) were significantly associated with a lower proportion of daytime SpO_2_ values below 90% (Additional file 1: Data 1). Multivariate analysis identified only PaO_2_ as a significant predictor of the proportion of daytime SpO_2_ values below 90% (β = -0.21, *p* = 0.04) (Additional file 1: Data 2).

**Figure 1:**
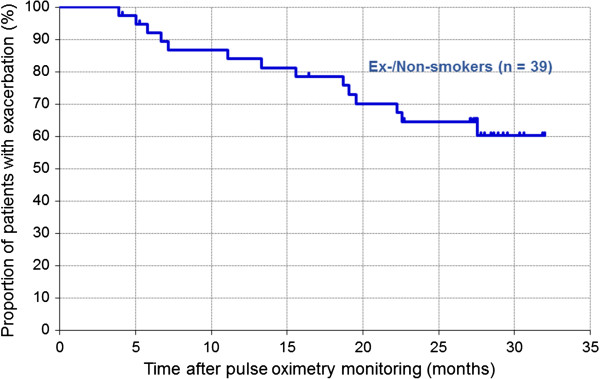
Kaplan–Meier curves for exacerbation of COPD in 39 ex-/non-smokers (blue line).

Five ex-smokers were lost to follow-up. Two ex-smokers died of acute heart failure and unknown cause, respectively. Thirty-two ex-/non-smokers were alive and still followed up at the time of data cut-off. One third of the patients experienced exacerbations during the follow-up period (Table 3). The median time from oximetry monitoring to the first exacerbation was not reached (Figure 1). Univariate (Table 4) and multivariate (Table 5) analyses did not detect any factors that were significantly associated with exacerbation.

**Table 3:** Characteristics of exacerbations in ex-/non-smokers (n = 39)

Follow-up duration (months)	Mean ± SD	26.4 ± 6.7
	Median (range)	27.8 (4.1–32.8)
All exacerbations	Total exacerbations/patients	21/13
Exacerbation-related admission	Total admissions/patients	13/9
Antibiotic use	Total uses/patients	15/10
Systemic steroid use	Total uses/patients	4/4

SD, Standard deviation.

**Table 4:** Univariate Cox proportional hazards analysis of relationships between variables and exacerbation in ex-/non-smokers (n = 39)

**Variable**	**Risk ratio**	**95% CI**	***p***
Age (years)	1.04	0.97 – 1.10	0.26
Unadjusted Charlson Comorbidity Index	0.93	0.57 – 1.51	0.76
Age-adjusted Charlson Comorbidity Index	1.06	0.76 – 1.46	0.74
Pack/years	1.00	0.98 – 1.01	0.69
Body mass index	0.87	0.73 – 1.03	0.10
mMRC dyspnoea grade	1.19	0.73 – 1.96	0.49
CAT score	1.08	1.00 – 1.16	0.054
PaO_2_	0.97	0.92 – 1.03	0.35
PaCO_2_	0.95	0.84 – 1.08	0.45
FEV_1_	0.60	0.23 – 1.53	0.28
%FEV_1_	0.99	0.96 – 1.02	0.45
Daytime			
Proportion of SpO_2_ values < 90% (%)	1.01	0.92 – 1.11	0.78
Mean heart rate	1.02	0.97 – 1.07	0.42
Nighttime			
Proportion of SpO_2_ values < 90%	0.98	0.92 – 1.03	0.41
3%ODI	0.92	0.82 – 1.02	0.12
Mean heart rate	1.01	0.95 – 1.07	0.71

CAT, Chronic obstructive pulmonary disease assessment test; CI, Confidence interval; FEV_1_, Forced expiratory volume in one second; mMRC, Modified Medical Research Council; ODI, Oxygen desaturation index; PaCO_2_, Partial pressure of arterial carbon dioxide; PaO_2_, Partial pressure of arterial oxygen; SpO_2_, Oxygen saturation by pulse oximetry.

**Table 5:** Multivariate Cox proportional hazards analysis of relationships between variables and exacerbation in ex-/non-smokers (n = 39)

**Variable**	**Risk ratio**	**95% CI**	***p***
Age-adjusted Charlson Comorbidity Index	0.96	0.67 – 1.37	0.82
Body mass index	0.88	0.72 – 1.06	0.18
CAT score	1.08	0.99 – 1.18	0.10
%FEV_1_	0.99	0.95 – 1.02	0.45
Daytime proportion of SpO_2_ values < 90%	1.00	0.84 – 1.18	0.97
Nighttime proportion of SpO_2_ values < 90%	0.99	0.92 – 1.05	0.68

CAT, Chronic obstructive pulmonary disease assessment test; CI, confidence interval; FEV_1_, forced expiratory volume in one second; SpO_2_, Oxygen saturation by pulse oximetry.

## Discussion

This study of Japanese COPD outpatients not receiving supplemental oxygen therapy included no daytime desaturators and few nighttime desaturators. The proportions of daytime and nighttime SpO_2_ values below 90% did not predict exacerbation.

To the best of our knowledge, only four previous studies have reported the results of continuous 24-hour oxygen saturation monitoring during activities of daily living using a portable pulse oximeter (Table 6) [3,5,11,18]. The population of the present study differed from the populations of these previous studies in terms of race, disease severity (%FEV_1_ and resting PaO_2_), physique (BMI), and SpO_2_ values. Patients with more severe COPD generally tend to be more underweight [19-21]. However, the patients in this study had milder COPD but were more underweight than patients in previous non-Asian studies. Additionally, our oximetry results were much better than in previous studies. Patients in this study had less frequent daytime and nighttime desaturations than those in the previous studies. Although even the nine patients with a resting PaO_2_ of 60–70 mmHg had better oxygen saturation profiles than patients in the previous non-Asian studies, their desaturation profiles and pulmonary function were comparable to those of the 55 non-desaturators in the study by Casanova *et al*., which also found that non-desaturators were less hypercapnic and less hypoxemic during the daytime than desaturators in spite of having similar pulmonary function and Saint George’s Respiratory Questionnaire scores [11]. Our nine patients with a resting PaO_2_ of 60–70 mmHg had lower resting PaCO_2_ values than patients in the previous studies. These comparisons suggest that desaturators are likely to have hypercapnic respiratory failure.

**Table 6:** Studies of continuous 24-hour oximetry monitoring during activities of daily living

**Authors(Year)[Ref]**	**N**	**PaO**_**2 **_**inclusion criteria**	**Supplemental oxygen therapy**	**PaO**_**2**_**PaCO**_**2**_**(mmHg)**	**%FEV**_**1**_	**BMI**	**Proportion of desaturators**	**Mean proportion of SpO**_**2 **_**values < 90%**
Soguel Schenkel (1996) [18]	30 inpts	Not defined	3 Patients*	Median (range)	Median (range)	Median (range)	Not described	Not described
				68 (54–89)	37 (16–64)	25.5 (17.1–42.5)		
				42 (35–51)				
Casanova (2006) [11]	88 outpts	60–70 mmHg	None	Mean ± SD	Mean ± SD	Mean ± SD	Daytime 22%	Non-desaturators
				64.7 ± 2.8	38 ± 13	27 ± 4	Nighttime 50%	24-hour 12.2%
				45 ± 5.7				Daytime 8.9%
								Nighttime 18.8%
								Desaturators
								24-hour 55.1%
								Daytime 42.3%
								Nighttime 77.3%
Garcia-Talavera (2008) [3]	67 outpts	60–70 mmHg	None	Median (range)	Median (range)	Not described	24-hour 30%	24-hour 17%
				66 (60–70)	37 (16–64)		Daytime 25%	Daytime 15%
				45 (32–57)			Nighttime 45%	Nighttime 23%
Trauer (2012) [5]	35 outpts	56–70 mmHg	None	Mean ± SD	Mean ± SD	Mean ± SD	24-hour 54%	24-hour 41.9%
				64.1 ± 4.5	37.5 ± 13.2	27.1 ± 7.0	Daytime 40%	Daytime 30.9%
				44.2 ± 6.0			Nighttime 77%	Nighttime 60.6%
This study	39 outpts	Not defined	None	Mean ± SD	Mean ± SD	Mean ± SD	Daytime 0%	Daytime 3.0%
				78.2 ± 8.9	50.9 ± 18.5	23.1 ± 3.8	Nighttime 8%	Nighttime 7.4%
				39.1 ± 4.9				
	9 outpts**	60–70 mmHg	None	66.2 ± 3.0	37.0 ± 14.6	23.9 ± 4.8	Daytime 0%	Daytime 8.4%
				38.0 ± 5.4			Nighttime 22%	Nighttime 20.2%

*Three patients had not previously received supplemental oxygen therapy, but started nocturnal oxygen therapy during admission to a pulmonary rehabilitation programme.

**Nine patients in this study had more severe COPD with mild-to-moderate hypoxaemia (PaO_2_ 60–70 mmHg).

BMI, body mass index; FEV_1,_ Forced expiratory volume in one second; Inpts, inpatients; Outpts, outpatients; PaO_2_, partial pressure of arterial oxygen; Ref, Reference; SD, Standard deviation; SpO_2_, Oxygen saturation by pulse oximetry.

In this study, oximetry results did not predict exacerbation. This is consistent with the findings of Trauer *et al*., who reported that ambulatory oximetry results in patients with a resting PaO_2_ of 56–70 mmHg did not predict exacerbation or survival [12]. They proposed four reasons for the lack of association between oximetry results and survival: 1) oximetry results are not fundamentally important for predicting prognosis, 2) progression of COPD is unpredictably heterogeneous, 3) the sample size was not large enough to detect differences, and 4) low ambulatory oxygen saturation reflected greater levels of physical activity. In the present study, the lack of association between oximetry results and the risk of exacerbation may be related to the small sample size, as the patients had mild COPD and therefore had lower rates of exacerbation and death than in the study by Trauer *et al.*[12]. However, the CAT score may be an independent predictor of exacerbation, as also recently reported in other studies [22,23].

This study has some limitations. First, despite using an activity log, it was not possible to accurately determine the patients’ physical activities at specific times. It is difficult for patients to accurately record activities by the minute, or accurately recall activities over longer periods of time [24]. Most patients in this study had inaccurate recordings, and patients frequently forgot to record any activities at all. The resulting activity logs were not useful for comparison with pulse oximetry results. It has been reported that patients with COPD are markedly inactive in daily life [25], and that the reduction in physical activity starts in the early stages of the disease, even before COPD is diagnosed [26]. It is therefore possible that the minimal desaturations recorded in this study reflected reduced activity levels. Use of motion sensors such as accelerometers seems to be a promising alternative to patient-reported activity logs. Only one previous study reported concomitant measurement of SpO_2_ and physical activity by oximeter, accelerometry and actigraphy, respectively [27]. However, the analysis in this study divided activities into only four categories: walking, slow-intermittent-walking, active-not-walking, and resting. These broad classifications are not sufficient for linking desaturation profiles with activities of daily living. Second, the influence of COHb on SpO_2_ values in the 12 current smokers was not assessed, because the COHb concentrations were not measured. Therefore, we excluded the current smokers from our analyses, and it remains unknown whether ambulatory oximetry monitoring is reliable in smokers. Finally, oximetry monitoring was performed only once, as in the previous studies. Only the study by Casanova *et al*. reported repeat 24-hour oximetry monitoring, at 1–3 weeks after the initial monitoring in 11 patients [11]. They did not find significant differences between the results of the two monitoring periods. Although it is necessary to investigate whether a single 24-hour monitoring period is sufficient for evaluation of the desaturation profile, it is difficult to perform monitoring over multiple periods because of patient discomfort and disturbance of activities of daily living while the monitor is attached.

## Conclusions

Our 24-hour ambulatory oximetry monitoring provided precise data regarding the desaturation profiles of Japanese outpatients with COPD. Both daytime and nighttime desaturations were infrequent. The proportion of SpO_2_ values below 90% did not predict exacerbation of COPD.

## Availability of supporting data

The data sets supporting the results of this article are included within the article and its additional files.

## Competing interests

The authors declare that they have no competing interests.

## Authors’ contributions

SM analysed the data and drafted the manuscript. SY designed the study. All authors contributed to conducting the study and collecting the data, and read and approved the final manuscript.

## Supplementary Material

Additional file 1 **Data 1.** Simple regression analysis of relationships between variables and the proportion of daytime SpO_2_ values below 90% (ex-/non-smokers, n = 39). **Data 2.** Multiple regression analysis of relationships between variables and the proportion of daytime SpO_2_ values below 90% (ex-/non-smokers, n = 39). Click here for file
