# Supplementary material for: Ambulatory pulse oximetry monitoring in Japanese COPD outpatients not receiving oxygen therapy
Source: Multidiscip Respir Med. 2014 Apr 17;9(1):24. doi: 10.1186/2049-6958-9-24 (PMC4021057; doi:10.1186/2049-6958-9-24)
Supplement: Additional file 1 — Data 1. Simple regression analysis of relationships between variables and the proportion of daytime SpO2 values below 90% (ex-/non-smokers, n = 39). Data 2. Multiple regression analysis of relationships between variables and the proportion of daytime SpO2 values below 90% (ex-/non-smokers, n = 39). [file 2049-6958-9-24-S1.docx]

**Supporting Data 1. Simple regression analysis of relationships between variables and the proportion of daytime SpO_2_ values below 90% (ex-/non-smokers, n = 39)**

|  | β | 95% CI | R^2^ | *p* |
| --- | --- | --- | --- | --- |
| Age (years) | 0.09 | −0.12 – 0.30 | 0.02 | 0.38 |
| Body mass index | −0.08 | −0.57 – 0.42 | 0.003 | 0.76 |
| mMRC dyspnoea grade | 2.38 | 0.90 – 3.85 | 0.22 | 0.002 |
| CAT score | 0.18 | −0.08 – 0.44 | 0.05 | 0.16 |
| PaO_2_ | −0.30 | −0.48 – −0.12 | 0.24 | 0.002 |
| PaCO_2_ | 0.14 | −0.25 – 0.52 | 0.01 | 0.48 |
| %FEV_1_ | −0.12 | −0.21 – −0.03 | 0.16 | 0.01 |

β, Unstandardized regression coefficient of the independent variable; CAT, Chronic pulmonary obstructive disease assessment test; CI, Confidence interval; FEV_1_, Forced expiratory volume in one second; mMRC, Modified Medical Research Council; PaCO_2_, Partial pressure of arterial carbon dioxide; PaO_2_, Partial pressure of arterial oxygen; R^2^, Coefficient of determination; SE, Standard error; SpO_2_, Oxygen saturation by pulse oximetry.

**Supporting Data 2. Multiple regression analysis of relationships between variables and the proportion of daytime SpO_2_ values below 90% (ex-/non-smokers, n = 39)**

|  | β | 95% CI | SE | *p* |
| --- | --- | --- | --- | --- |
| mMRC dyspnoea grade | 1.81 | −0.29 – 3.90 | 1.03 | 0.09 |
| CAT score | −0.12 | −0.40 – 0.17 | 0.14 | 0.40 |
| PaO_2_ | −0.21 | −0.41 – 0.01 | 0.10 | 0.04 |
| %FEV_1_ | −0.03 | −0.14 – 0.08 | 0.05 | 0.60 |

The coefficient of determination (R^2^) was 0.34 for ex-/non-smokers.

β, Unstandardized regression coefficient of the independent variable; CAT, Chronic pulmonary obstructive disease assessment test; CI, Confidence interval; FEV_1_, forced expiratory volume in one second; mMRC, Modified Medical Research Council; PaO_2_, Partial pressure of arterial oxygen; SE, Standard error; SpO_2_, Oxygen saturation by pulse oximetry.
